# The impact of Covid-19 vaccines on fertility-A systematic review and metanalysis: Emanuele La Gatta

**DOI:** 10.1093/eurpub/ckac131.415

**Published:** 2022-10-25

**Authors:** D Zaçe, E La Gatta, L Petrella, A Shukhovtseva, ML Di Pietro

**Affiliations:** 1 Università Cattolica del Sacro Cuore, Rome, Italy; 2 Department of Life Sciences and Public Health, Università Cattolica, Rome, Italy; 3 Sechenov University, Moscow, Russia

## Abstract

**Introduction:**

Despite literature’s proofs about their safety, concerns arose regarding adverse events due to Covid-19 vaccines, including the possible impact on fertility, accentuated by misinformation and anti-vaccine campaigns. The aim of this study was to evaluate the Covid-19 vaccines’ impact on male and female fertility.

**Methods:**

PubMed, Scopus, Web of Science, Cochrane and Embase databases were searched for eligible studies until March 7th, 2022. Primary studies investigating the Covid-19 vaccines impact on male and female fertility, were included. Studies’ quality was assessed by the Newcastle-Ottawa and the Before and After Quality Assessment scales for cohort and pre-post studies, respectively. Random-effect meta-analyses were performed for parameters considered in ≥ 2 studies, calculating means, p-values and 95% Confidence Intervals (CIs). I^2^ statistics was used to assess statistical heterogeneity.

**Results:**

Out of 1406 studies screened, 20 studies were included in the systematic review. These studies, conducted in Israel (35%), USA (30%), Russia (25%), China (5%) and Italy (5%), were of poor (15%), moderate (75%) and good (10%) quality. Meta-analyses among five studies considering several vaccines were performed for pre- and post-vaccination sperm progressive motility ((49%, 95% CI 36-67% vs 49%, 95% CI 39-61%; p = 0.963) and concentration (64.39 mln/ml, 95% CI 47.51-87.28 and 72.00 mln/ml, 95% CI 51.22-101.21; p = 0.03). Subgroup meta-analyses based on the type of vaccine showed no significant difference: between vaccinated with mRNA vaccines and non-vaccinated regarding biochemical pregnancy rates; pre- and post-vaccination with Gam-COVID-Vac regarding testosterone, FSH and LH levels; pre- and post-vaccination with BNT162b2 vaccines regarding sperm volumes.

**Discussion:**

There is no scientific proof of any association between Covid-19 vaccines and infertility in men or women. Misinformation and doubts about vaccines should be properly addressed.

**Key messages:**

• The doubts regarding Covid-19 vaccines’ impact on both male and female fertility resulted to be unfounded. Covid-19 vaccines remain the most important weapon to fight the pandemic.

• It is important to keep providing to public opinion and health care providers evidence-based scientific information, in order to effectively combat misinformation and anti-vaccines campaigns.

